# Impacts of the Three Gorges Dam on microbial structure and potential function

**DOI:** 10.1038/srep08605

**Published:** 2015-02-27

**Authors:** Qingyun Yan, Yonghong Bi, Ye Deng, Zhili He, Liyou Wu, Joy D. Van Nostrand, Zhou Shi, Jinjin Li, Xi Wang, Zhengyu Hu, Yuhe Yu, Jizhong Zhou

**Affiliations:** 1 State Key Laboratory of Freshwater Ecology and Biotechnology, Institute of Hydrobiology, Chinese Academy of Sciences, Wuhan, China; 2 Institute for Environmental Genomics and Department of Microbiology and Plant Biology, University of Oklahoma, Norman, OK, USA; 3 State Key Joint Laboratory of Environment Simulation and Pollution Control, School of Environment, Tsinghua University, Beijing, China; 4 Earth Sciences Division, Lawrence Berkeley National Laboratory, Berkeley, CA, USA; 5 CAS Key Laboratory of Environmental Biotechnology, Research Center for Eco-Environmental Sciences, Chinese Academy of Sciences, Beijing, China; 6 Graduate University of Chinese Academy of Sciences, Beijing, China

## Abstract

The Three Gorges Dam has significantly altered ecological and environmental conditions within the reservoir region, but how these changes affect bacterioplankton structure and function is unknown. Here, three widely accepted metagenomic tools were employed to study the impact of damming on the bacterioplankton community in the Xiangxi River. Our results indicated that bacterioplankton communities were both taxonomically and functionally different between backwater and riverine sites, which represent communities with and without direct dam effects, respectively. There were many more nitrogen cycling *Betaproteobacteria* (e.g., *Limnohabitans*), and a higher abundance of functional genes and KEGG orthology (KO) groups involved in nitrogen cycling in the riverine sites, suggesting a higher level of bacterial activity involved in generating more nitrogenous nutrients for the growth of phytoplankton. Additionally, the KO categories involved in carbon and sulfur metabolism, as well as most of the detected functional genes also showed clear backwater and riverine patterns. As expected, these diversity patterns all significantly correlated with environmental characteristics, confirming that the bacterioplankton communities in the Xiangxi River were really affected by environmental changes from the Three Gorges Dam. This study provides a first comparative metagenomic insight for evaluating the impacts of the large dam on microbial function.

As one of the most abundant types of organisms on Earth, bacterioplankton have been found almost anywhere water exists^1^. They are an extremely diverse and highly dynamic group whose activities directly influence the water conditions in their immediate environment as well as having profound effects on biospheric biogeochemical cycling^2^. In turn, bacterioplankton communities also undergo significant shifts in composition or abundance at both spatial and temporal scales in response to environmental variations^3^,^4^,^5^. Construction of river dams can significantly alter river hydrology and aquatic ecology upstream and downstream of the dam^6^, creating environments that have been significantly altered physically, chemically and biologically. These changes can, in turn, greatly affect the aquatic microorganisms living in the reservoir systems, especially environmentally sensitive organisms, such as bacterioplankton^7^,^8^,^9^. However, most of the investigations on the effects of dams to planktonic organisms has focused on community composition and has primarily relied on morphological methods for examining community changes. Fortunately, the recently developed culture-independent rRNA gene sequencing has already offered a useful glimpse into microbial diversity^10^,^11^,^12^,^13^,^14^, and the more powerful high-throughput metagenomic sequencing technology is a further key innovation for exploring community composition and function in microbial ecology^15^. However, knowledge regarding microbial function in response to dam impacts is still lacking.

We know that similar communities may have strikingly divergent functions and distinct community configurations can perform similar functions^16^. Consequently, an understanding of the community composition is insufficient to reveal the functional significance of a community in a particular environment. Recently, the whole metagenomic shotgun sequencing and high-throughput GeoChip functional gene microarray analysis have become attractive approaches to examine the functional properties of microbial communities^17^,^18^,^19^. For aquatic environments, most attention has focused on ocean environments^14^,^20^ and a few freshwater lakes^15^,^21^, but very little attention has been paid to bacterioplankton metagenomes in river/reservoir systems^22^. The Xiangxi River is an ideal river/reservoir system to address the impacts of dams on bacterioplankton since it is the nearest large tributary to the Three Gorges Dam. It has clear backwater and riverine areas, which represent bacterioplankton communities with or without direct dam effects, respectively.

Since the impoundment of the Three Gorges Reservoir began in June 2003, the backwater area of the Xiangxi River (the region with water being held or pushed back by the dam) has been transformed from a riverine system into a lacustrine system, accompanied by significant ecological and environmental changes. For example, the water level has increased more than 40 m; the water flow velocity has dropped significantly^23^; and water blooms have been detected in spring at some newly formed bays^24^,^25^. Although phytoplankton succession and the process underlying blooms in the Xiangxi River have been frequently studied^24^,^25^,^26^, our understanding of damming effects on planktonic microorganisms is largely restricted to phytoplankton shifts and related environmental changes, while changes in bacterioplankton have been largely ignored. A few studies have addressed the bacterial community composition along the Yangtze River using more course-level culture-independent methods such as PCR-DGGE and clone libraries^8^,^27^,^28^; but high-throughput sequencing and functional profile-based approaches have not been involved. As such, there are still questions regarding the structural and functional responses of bacterioplankton to environmental changes caused by the Three Gorge Dam and the biogeochemical roles they play in this newly formed reservoir system.

To gain insight into the bacterioplankton community structure and the functional potential of this community in the Xiangxi River after the damming and to better understand bacterioplankton roles in biogeochemical cycling, we performed 16S rRNA gene sequencing, functional gene array (GeoChip 5.0) analysis, and metagenomic shotgun sequencing on six bacterioplankton communities from different areas of the Xiangxi River. Specifically, we addressed the following questions in this study: (i) are the bacterioplankton communities in backwater and riverine sites taxonomically and/or functionally different due to effects from the dam; (ii) what is the relationship between the bacterioplankton community structure (both phylogenetic and functional) and the environmental conditions changed by the damming regulation; and (iii) how is the community functional potential altered by the dam, specifically those functions involved in the cycling of key natural elements (e.g., carbon, nitrogen and sulfur)?

## Results

### Environmental characteristics, phyto- and zooplankton composition

**Figure 1:**
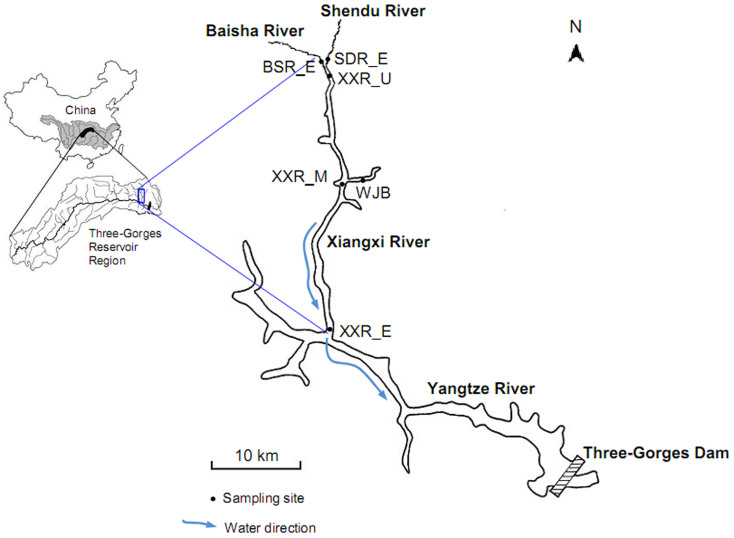
Location of the six sampling sites along the Xiangxi River in the Three Gorges Reservoir (created using Adobe Photoshop 8.01 software). The estuary, midstream and upstream sites along the Xiangxi River are abbreviated as XXR_E, XXR_M, and XXR_U, respectively; the other three sites are the Wujia Bay (WJB), the estuary of the Baisha River (BSR_E) and the estuary of the Shendu River (SDR_E).

Physicochemical analysis indicated that the nutrient concentrations of total phosphorus (TP), phosphate phosphorus (P-PO_4_) and chemical oxygen demand (COD) were significantly lower in the backwater sites than in the riverine sites (*t*-test, *P* < 0.05). However, the concentrations of total nitrogen (TN), ammonium nitrogen (N-NH_4_) and nitrate nitrogen (N-NO_3_) were significantly higher in the backwater area (Table S1). These nutrient variations, which have been shown to be significantly associated with bacterioplankton^28^,^29^, could be directly or indirectly affected by the Three Gorges Dam. However, some other factors such as pH, turbidity, transparency, temperature, dissolved oxygen, and conductivity showed no statistical difference between the two investigated areas (Table S1) as showed in Figure 1.

Based on morphological identification (generally to the genus or species level), *Cyclotella* sp. were the most abundant algae (10^8^–10^9^ ind. L^−1^) in the Xiangxi River (Table S2). Although only *Pyrrophyta* and *Pandorina* spp. abundances showed significant difference between backwater and riverine sites (*t*-test, *P* < 0.05), the algal diversity in riverine sites was much higher than in the backwater sites (Table S2). There were many more zooplankton taxa (especially protozoa and rotifer) in the riverine sites (Table S2). Furthermore, the riverine sites generally harbored a significantly higher abundance of rotifer species (e.g., *Trichocerca pusilla*, *Polyarthra vulgaris*, *Synchaeta stylata*, *Anuraeopsis fissa*) and *Ciliophora*. However, there were more large zooplanktonic cladocerans (e.g., *Daphnia hyaline, Bosmina longirostris*) and copepods (e.g., *Sinocalanus dorrii*) in the backwater sites than in the riverine sites (Table S2).

### Bacterioplankton composition determined using high-throughput sequencing

The valid sequences obtained could be assigned to 1,368 operational taxonomic units (OTUs) using the UClust method. The overlap of detected OTUs between backwater and riverine sites (33.71% ± 1.55%) was much lower than that observed within the backwater or riverine sites (54.98% ± 3.83%, one-way ANOSIM, *P* = 0.0006). These OTUs clustered into 22 phyla, 51.17% of which were classified as *Proteobacteria* (Fig. S1), followed by the *Bacteroidetes* (22.22%) and *Actinobacteria* (15.28%). Among all the 18 genera that each accounted for more than 1% of the total bacterial abundance, 8 showed significant differences between the backwater and riverine sites (mainly from *Bacteroidetes* and *Proteobacteria*, Table S3.1). Among all dominant OTUs (each > 1% of the total bacterial abundance), more than a half had abundances that were significantly different between the backwater and riverine sites (mainly from *Proteobacteria* and *Actinobacteria*, Table S3.2). Rarefaction analysis indicated that sequencing for all of the communities investigated here reached near saturation at a genetic distance of 3% (Fig. S2).

The Chao value, Shannon, inverse Simpson richness, Pielou and Simpson evenness all indicated that the α-diversity of bacterioplankton was significantly higher (*t*-test, *P* < 0.01) in the backwater sites than in the riverine sites (data not shown). The β-diversity also suggested that bacterioplankton communities within the backwater/riverine sites were much more similar than between the backwater and riverine sites. Although the Bray-Curtis distance based dissimilarity tests already confirmed that the community differences between the two investigated areas were significant (PERMANOVA, *F* = 0.755, *P* = 0.001). We further applied the Raup-Crick probability-based index (*S_RC_*)^30^ to confirm whether differences among the communities were significant (*S_RC_* < 0.05), or due to chance (0.05 < *S_RC_* < 0.95). The *S_RC_* provides a measure of statistically significant similarity and dissimilarity at the 95% confidence level, and therefore is less affected by sampling bias^31^. An additional feature of this index is that biogeographic data are weighted on the basis of the frequency of occurrence, and widespread taxa do not disproportionately influence the similarity^32^. We found that all *S_RC_* values between the backwater and riverine sites were less than 0.05, confirming that the backwater communities were significantly different from those in the riverine sites. But all *S_RC_* values within the backwater or riverine sites were larger than 0.95 (significantly similar).

**Figure 2:**
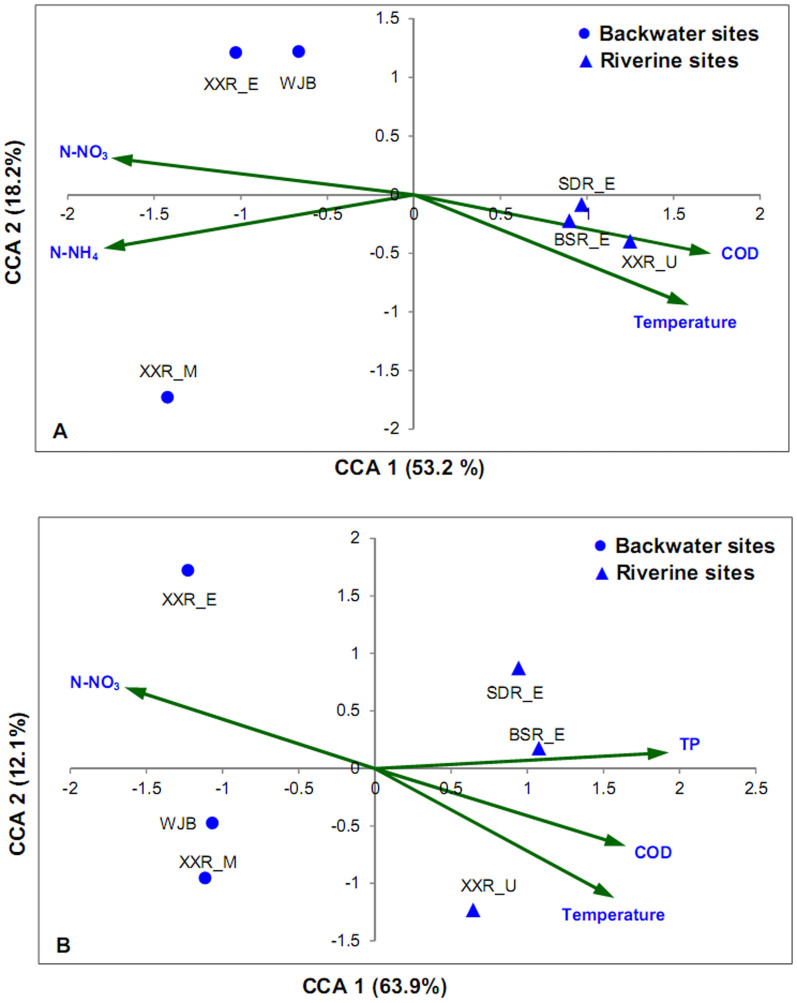
Canonical correspondence analysis (CCA) shows the relationships between environmental variables and the bacterial OTUs (A) or functional genes (B). Only variables that were significantly correlated with the community (forward selection with Monte Carlo test, *P* < 0.05) are shown. Abbreviations: TP, total phosphorus; N-NH_4_, ammonium nitrogen; N-NO_3_, nitrate nitrogen; COD, chemical oxygen demand. The full name of each sampling site is shown in Figure 1.

**Figure 3:**
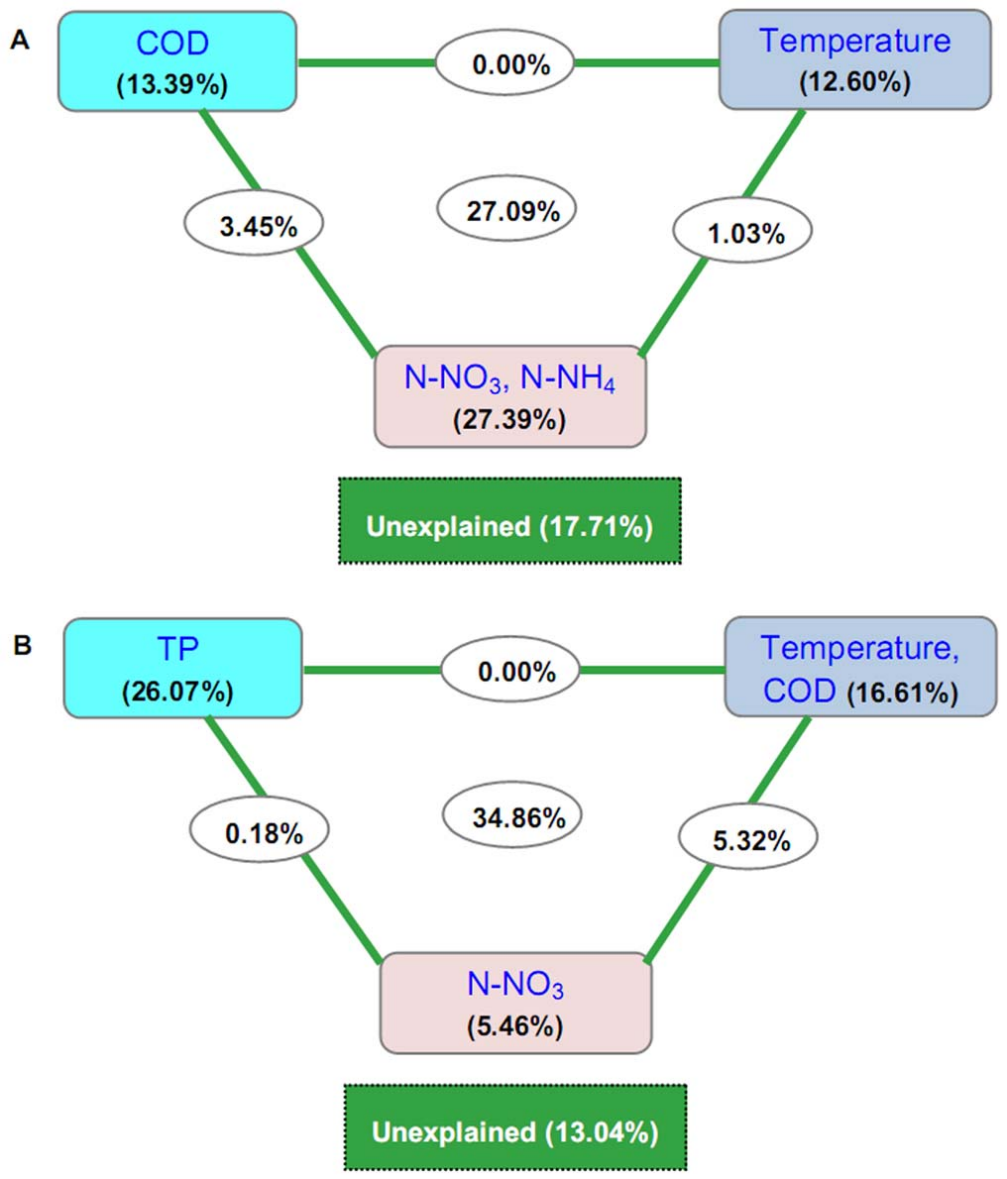
Variance partitioning canonical correspondence analysis (CCA) shows the relative effects of multiple variables on the composition of bacterial taxa (A) and functional genes (B). The squares represent the effect of individual variables by partitioning out the effects of the other variables. The ellipses between the squares represent the combined effects from the variables on either side of the ellipse. The combined effects of all variables are shown by the ellipse in the center. The square at the bottom of each figure represents the effect that could not be explained by any of the variables tested. Abbreviations: TP, total phosphorus; N-NH_4_, ammonium nitrogen; N-NO_3_, nitrate nitrogen; COD, chemical oxygen demand.

In terms of relative abundance, *Proteobacteria* (mainly *Betaproteobacteria*) was the most abundant bacterial phylum in the Xiangxi River (accounting for 32.93%–74.67% among different sites), and had a significantly higher abundance in the riverine sites than in the backwater sites (Table S4). *Actinobacteria* was significantly higher in the backwater sites (18.78%–34.75%) than in the riverine sites (Table S4). The canonical correspondence analysis (CCA) indicated that the N-NH_4_, N-NO_3_, COD, and temperature showed significant relationships with taxonomic composition (Fig. 2A). The first two axes explained 71.4% of the taxonomic information. Variance partitioning CCA further suggested that nitrogen sources of N-NH_4 _and N-NO_3_ may be the most important factors affecting taxonomic composition (Fig. 3A).

### Functional gene diversity detected using GeoChip 5.0

Microarrays detected significantly more probes (*t*-test, *P* < 0.05) in the riverine sites communities (22,880 ± 680) than in the backwater sites (18,022 ± 830). The overlap of detected probes within backwater/riverine sites (90.46% ± 3.88%) was much higher than that between samples from backwater and riverine sites (73.26% ± 2.81%, one-way ANOSIM, *P* = 0.0001). The 74 most abundant functional genes (sum of normalized signal intensities > 100) were all significantly different between the backwater and riverine sites (Table S5). Generally, profiles derived from the riverine sites showed significantly higher gene signal intensities than those derived from the backwater sites (*t*-test, *P* < 0.05). Moreover, 72.14% of all detected functional genes were significantly different between the communities derived from backwater and riverine sites, which was confirmed by dissimilarity testing (PERMANOVA, *F* = 0.807, *P* = 0.001).

For each gene category (e.g., carbon, nitrogen, phosphorus, and sulfur cycling; metal homeostasis; organic remediation; secondary metabolism; and virulence), significantly fewer probes showed positive signals in samples from backwater sites than those from riverine sites (*t*-test, *P* < 0.01). Significant differences were further confirmed by comparing normalized signal intensities for each gene category. Similar results were obtained at the sub-category level, with the exception of nitrogen assimilation genes (Table S6). CCA analysis indicated that TP, N-NO_3_, COD, and temperature were significantly associated with microbial functional diversity. The first two axes explained 76.0% of the functional information (Fig. 2B). TP was the most important environmental factor affecting functional diversity (Fig. 3B).

### Functional characteristics determined using metagenomic shotgun sequencing

Metagenomic shotgun sequencing generated 211 million raw reads (31–41 million per sample) with a total of 42 Gb for all six samples (6.4–8.1 Gb per sample). Most of reads (96.6%) passed our strict quality control requirements. As sequence depth can affect estimation of the relative abundances of gene categories^33^, all shotgun datasets were rarefied to the same amount of reads via random resampling before downstream analysis. The valid sequences were assembled into contigs with a total length of 37.1–143.0 Mbp for different samples (N50 = 942 ± 105 bp), and 48,789–219,956 open reading frames (ORFs, > 500 bp) were annotated and matched using the KEGG and eggNOG databases. Finally, a total of 4,024 KEGG orthology (KO) groups involved in various pathways were annotated. Although there were no significant differences (*t*-test, *P* > 0.05) in the number of detected ortholog groups, the samples clustered into two groups based on relative abundances (Fig. S3) and indicated clear differences between backwater and riverine sites in the Xiangxi River. The KEGG ortholog groups involved in the carbon, nitrogen and sulfur metabolic pathways showed similar patterns (Fig. S4, S5, S6).

Although only two COG functional categories (cytoskeleton, and RNA processing and modification) were significantly different between the backwater and riverine sites (Table S7), in generally most of the categories related to core metabolic functions (e.g., energy production and conversion, amino acid, coenzyme, lipid and nucleotide transport and metabolism, secondary metabolites biosynthesis, transport and catabolism) showed relatively higher abundance in the backwater sites than in the riverine sites.

## Discussion

It is well known that plankton are very sensitive to environmental variations, and therefore the physical and chemical changes associated with damming may have a detrimental effect on plankton. In addition, since plankton are at the bottom of the food chain, biological impacts of damming will present first effects on the plankton community as shifts in composition or abundance^9^. Water flow velocity, which is one of the most important factors affecting plankton in river systems^3^, can be significantly decreased by damming regulation. The Three Gorges Dam, constructed in the middle reach of the Yangtze River from 1994 and 2006, has significantly increased the Xiangxi River water level^23^ and decreased flow velocity in the main channel^7^. The effects of the water being held or pushed back (backwater) can be felt more than 30 km up the estuary of Xiangxi River. Six representative sites along this river, representing direct dam effects (i.e., backwater XXR_E, XXR_M and WJB) or just indirect dam influences (i.e., riverine XXR_U, BSR_E and SDR_E, Fig. 1) were studied. We hypothesized that the structural and functional patterns of communities directly impacted by the Three Gorges Dam would be significantly different from those indirectly affected.

16S rRNA gene sequencing results indicated that the backwater area showed higher bacterioplankton α-diversity than riverine sites (Fig. S2). This may be attributed to: (i) a greater abundance of protozoa and rotifer, and more zooplankton taxa in the riverine sites (Table S2) exerting a strong top-down effect on the bacterioplankton^31^,^34^; and/or (ii) the lake-like conditions in the backwater sites, characterized by low flow velocity, low turbidity and greater nitrogenous nutrients (Table S1), may support a greater variety of bacterioplankton lineages^7^,^9^. Similar to the bacterial community composition observed in other rivers^9^,^35^, *Betaproteobacteria* was present at a much higher proportion in upstream waters than in downstream waters, whereas *Actinobacteria* was at a much higher percentage in backwater sites (Table S4). These results support our hypothesis that the bacterial community composition was significantly impacted by damming and the associated environmental changes. This was further confirmed by the Mantel test (Table 1), and also in agreement with a recently reported study showing that bacterioplankton community structure was affected by a large dam in the Ebro River^9^.

**Figure 4:**
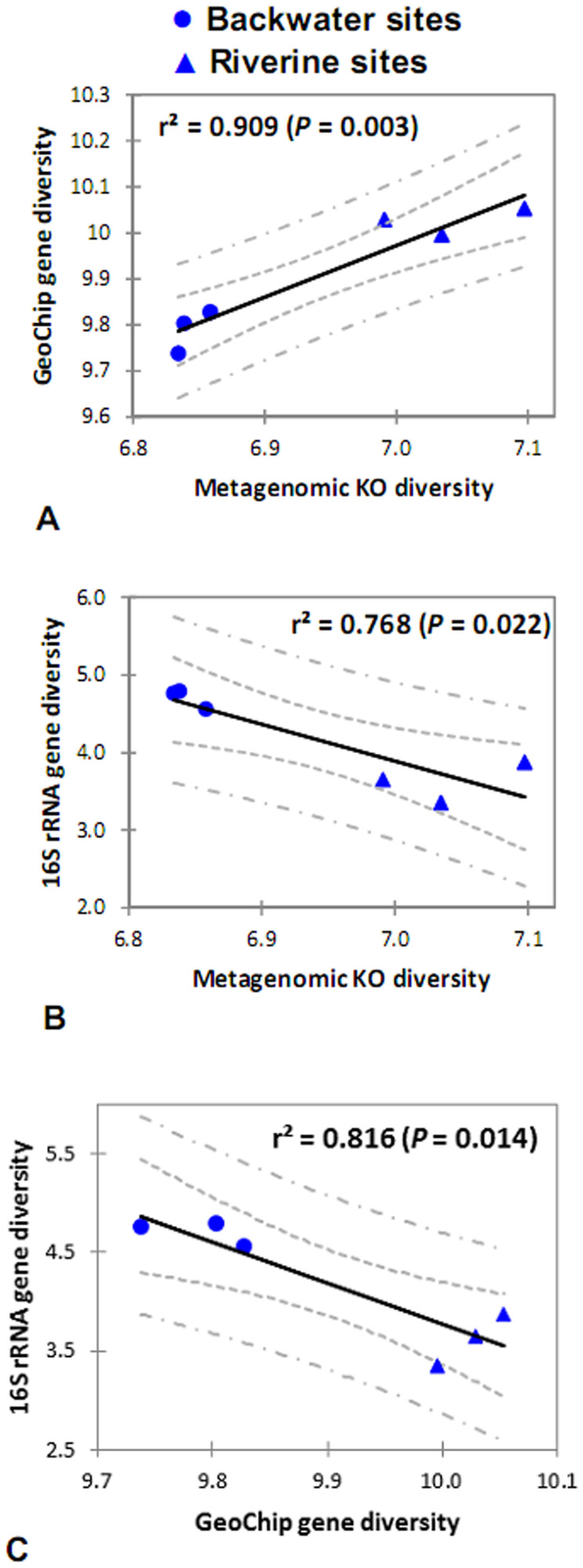
Pairwise comparison performed based on the α-diversity (Shannon index) determined using metagenomic shotgun sequencing (KO diversity), GeoChip 5.0 (gene diversity) and amplicon sequencing (16S rRNA gene diversity). The 95% confidence and prediction intervals are given (inner and outer dashed lines, respectively).

Next, we wanted to determine whether the compositional differences within the communities inevitably affected community-level function, particularly carbon, nitrogen, phosphorus and sulfur cycling. The functional gene-based GeoChip 5.0 used here detected a higher functional α-diversity than that calculated from OTU composition, suggesting that particular species show distinct functional potential in response to environmental variation. Although there is some debate about the use of functional α-diversity, it has been increasingly applied to both macro- and micro-organisms to evaluate the functional characteristics of targeted communities^36^,^37^,^38^,^39^. We found riverine communities showed significantly higher functional gene richness than backwater communities (*t*-test, *P* < 0.05), which is in almost complete contrast to the bacterioplankton taxonomic richness. The metagenomic KO diversity showed good agreement with the functional gene diversity and was also negatively correlated with taxonomic diversity (Fig. 4). These results suggest that taxonomic and functional analysis should be employed simultaneously for a better understanding of the microbial “black-box”, because community structure does not necessarily dictate functional diversity of a microbial community^40^.

Interestingly, 67.0% of the detected genes showed significant relationships to environmental factors, but only 11.2% of the taxonomic genera were significantly correlated with environmental conditions (Table 2). This seems to imply that functional gene patterns may be more sensitive to environmental variations than taxonomic composition. Similarly, functional genes appeared to be more appropriate than ‘species’ information in addressing questions regarding bacterial community assembly^16^. As bacterioplankton are essential players in the release of phosphorus^41^ and nitrogen fixation in aquatic ecosystems^29^, changes in functional genes involved in nitrogen and phosphorus cycling theoretically provide important implications to geochemical cycling. We found that TP and nitrogen sources (N-NH_4 _and N-NO_3_) were the most important factors affecting the bacterioplankton community function and composition, respectively (Fig. 3). The relationship between bacterioplankton and TP is frequently observed in freshwater systems^28^,^42^. The relationship between taxonomic composition and nitrogen sources is also not surprising as that was reported in some other river system such as Ebro River^9^. This correlation may be attributed to the higher abundance of *Proteobacteria* (Table S4), which includes many members involved in nitrogen cycling^29^,^43^. There really has high abundance of *Proteobacteria* OTUs/genera (e.g., *Limnohabitans*, *Acidovorax*, Table S3.1, S3.2) involved in nitrogen cycling. However, in-depth studies are needed to determine how the detected bacterial members/genes stimulate nutrient cycling processes in this ecosystem.

The metabolic potential and activity revealed by shotgun sequencing suggest that both the overall metabolic pathway (Fig. S3) and the specific biochemical processes (Fig. S4, S5, S6) reflect correlations between the bacterioplankton community and environmental variations caused be damming. For example, the lower reaches of the Xiangxi River have been transformed from a riverine system into a lacustrine system^28^,^44^. The resultant environmental changes have caused water blooms in some of the newly formed bays in the Xiangxi River^24^,^25^. The Mantel test further confirmed the significant relationship between KO groups and environmental factors (*r* = −0.564, *P* = 0.029, Table 1). Interestingly, 35.48% of the KO groups (Fig. S5, Table 3) involved in nitrogen cycling showed significant differences between the backwater and riverine sites. These significantly different KO groups were primarily involved in coding enzymes that catalyze the conversion of nitrate to ammonia (Fig. S7), which is in agreement with gene abundance data obtained from the GeoChip 5.0 (Fig. S8). The lower nitrogenous nutrients in the riverine sites (Table S1) could be associated with the higher abundance of algae, as nitrogen has been found to be a limiting nutrient for phytoplankton growth in the Xiangxi River^45^. The higher abundance of phytoplankton in the riverine sites during our investigating period (as determined by morphological counting and the concentration of Chl *a*) possibly consumed considerable nitrogenous nutrients, and therefore the concentrations of N-NH_4_, N-NO_3_, and TN were relatively low. This high nitrogenous requirement of phytoplankton may stimulate the bacteria to try and regenerate these nutrients by increasing populations containing the functional genes involved in nitrogen cycling, resulting in higher signal intensities of those genes in the riverine sites (Fig. S8). We should also acknowledge that there may have some other biogeochemical processes contributing to the differences of detected nitrogenous substances.

In summary, our results suggest that the Three Gorges Dam does significantly affect the structure and function of bacterioplankton communities in the Xiangxi River as shown by clear community patterns between sites with or without direct dam effects, which represented by backwater and riverine communities, respectively (Table 3). In addition, although the different methods used in describing the microbial diversity did not necessarily give identical results^33^,^46^, our results suggest that the three important culture-independent approaches used in this study detected similar bacterioplankton patterns along the Xiangxi River. Moreover, all of the diversity revealed by the different methods significantly correlated with the environmental factors. The nearest taxon index (NTI, all values larger than 2) also confirmed that environmental filtering was one of the major processes driving the bacterioplankton community changes in the Xiangxi River. This study not only provides a comprehensive metagenomic insight into the bacterioplankton community in the well-known Three Gorges Reservoir, but also enhances our understanding on the effects of damming on microbial community function.

## Methods

### Study area and sampling procedures

This study was conducted in the Xiangxi River, which lies 32 km upstream of the Three Gorges Dam, has a mainstream length of 94 km, a watershed area of 3,099 km^2^, and a natural fall of 1,540 m^47^. To address the possible impacts of the Three Gorges Dam on the bacterioplankton communities, we collected the samples from the estuary (XXR_E), midstream (XXR_M) and upstream of Xiangxi River (XXR_U), as well as Wujia Bay (WJB) and two secondary tributary sites at the estuary of Baisha River (BSR_E) and Shendu River (SDR_E) in April 2013 before the spring bloom. These sites can be classified into backwater (XXR_E, XXR_M and WJB) and riverine sites (XXR_U, BSR_E and SDR_E, Fig. 1). They were selected to represent a wide range of environmental gradients under the influences of the Three Gorges Dam, which may harbor various bacterioplankton communities and can be used to further determine how the functional capabilities are affected by a huge dam. In this study, we only examined bacterioplankton at a single time point per site, as it was not our goal to evaluate temporal variability.

At each site, equal volumes of water (10 L) were collected from the depths of 0.5, 5 and 10 m in the middle point of the river. After mixed fully in a bucket, water was split for the following different experiments. For DNA-based analysis, one liter of this mixed water was sequentially filtered through a 1.2-μm (Whatman, NJ, USA) and 0.22-μm filter paper (Millipore, MA USA) to collect most of the bacterioplankton organisms. The filtration was immediately performed onboard, and the filters were stored at −20°C until DNA extraction. Samples for morphological analysis were fixed with 1.5% Lugol's solution (final concentration), and after 24 h sedimentation, the above solution was removed by a siphon and the microplankton that had settled to the bottom were concentrated to 30 mL for abundance determination. Another set of samples was pre-processed based on standard methods^48^ for physicochemical analysis.

### Physicochemical analysis and morphological identification

Conductivity, dissolved oxygen (DO), turbidity, and pH were measured using a Professional Plus Multi-Parameter Probe (YSI, OH, USA) *in situ*. Temperature was read from a thermometer, and transparency was determined using a Secchi disc. Chemical characteristics, including chemical oxygen demand (COD), oxidation-reduction potential (ORP), total nitrogen (TN), ammonium nitrogen (N-NH_4_), nitrate nitrogen (N-NO_3_), total phosphorus (TP), and phosphate phosphorus (P-PO_4_) were measured using standard methods as described previously^48^. Chlorophyll *a* (Chl *a*) was extracted from a Whatman GF/C filter for 24 h with 90% acetone, centrifuged at 3,000 rpm for 10 min and then spectrophotometrically quantified^48^.

Zooplankton were identified and counted under an Axioplan 2 Imaging microscope (Zeiss, Jena, Germany) using a method described previously^4^. Phytoplankton analysis was performed under an optical microscope (Olympus BH-2, Tokyo, Japan) as previously described^25^.

### DNA extraction and GeoChip 5.0 analysis

The 1.2-μm and 0.22-μm filters used to collect microorganisms were used to extract DNA with the PowerWater® DNA Extraction kit (Mo Bio, CA, USA) separately according to the manufacturer's instructions. The DNA extracted from the two filters were pooled as a single sample and purified using a Genomic DNA Clean & Concentrator™ kit (Zymo, CA, USA) for downstream experiments. The purified DNA concentration was quantified using a PicoGreen dsDNA Assay kit (Invitrogen, CA, USA).

For each sample, 500 ng of DNA was labeled with the fluorescent dye Cy-3 (GE Healthcare, CA, USA) by random priming as described previously^49^; then purified using a QIAquick Purification kit (Qiagen, CA, USA) and then dried in a SpeedVac (Thermo Savant, NY, USA). The dried DNA was rehydrated with 13 μL of DNase/RNase-free distilled water, mixed completely, and then centrifuged to collect all liquid at the bottom of the tube. A total of 42 μL of buffer, including 1× HI-RPM hybridization buffer,1× aCGH blocking agent, 0.05 μg·μL^−1^ Cot-1 DNA, 10 pM universal standard, and 10% formamide (final concentrations), was added to each sample. After mixing completely, the solution was spun down and incubated at 95°C for 3 min, then incubated at 37°C for 30 min. The prepared samples were hybridized with GeoChip 5.0 arrays (60 K) at 67°C for 24 h with a rotation at 20 rpm in a hybridization oven. GeoChip 5.0 is the newest generation of GeoChip. The 60 K arrays used in this study contained approximately 60,000 probes targeting hundreds of gene families involved in various biogeochemical processes such as carbon, nitrogen, phosphorus, and sulfur cycling, metal homeostasis, organic remediation, secondary metabolism, and virulence. The scanned images of hybridized GeoChips were converted and extracted using Agilent Feature Extraction 11.5 software (Agilent Technologies, Inc., CA, USA); the extracted information was then analyzed using the microarray analysis pipeline on our web site (http://ieg.ou.edu/microarray/) as previously described^50^.

### MiSeq sequencing of 16S rRNA gene amplicons

The composition of bacterioplankton was analyzed using Illumina MiSeq sequencing of 16S rRNA gene amplicons. The V4 region of the 16S rRNA gene, which can yield accurate taxonomic information and shows few biases for various bacterial taxa^51^, was amplified with the primer set 515f (5′-GTGCCAGCMGCCGCGGTAA-3′)/806r 5′-GGACTACHVGGGTWTCTAAT-3′), and all PCR amplifications were conducted in triplicate for each sample. An initial 10 cycles of PCR amplification were performed. The products were then purified with Agencourt® Ampure® XP (Beckman Coulter, Inc., CA, USA) and used as a template for the second PCR amplification of 20 cycles using the same primer set; however, the reverse primer contained the appropriate adapters and different barcodes to distinguish samples. PCR products were visualized using 1% agarose gels stained with ethidium bromide, and negative controls were always performed to confirm the absence of contamination. True positive amplicons were quantified using a PicoGreen dsDNA Assay kit (Invitrogen, CA, USA), combined equally and then gel purified. The DNA library was sequenced at the Institute for Environmental Genomics using the Illumina MiSeq platform according to the manufacturer's instructions.

Quality filtering and the processing of MiSeq reads were conducted on our Galaxy pipeline. After trimming the primers and deleting sequences containing Ns, high quality sequences in length of 245–260 bp were kept for subsequent analysis. We ultimately obtained 18,256–24,012 high-quality sequences for different samples. To correct for differences in sequencing depth, 17,570 OTUs were randomly resampled for each sample before performing the downstream analysis. The taxonomic dissimilarities/similarities between each pair of communities were determined using the Bray-Curtis and Raup-Crick indexes.

### Metagenomic shotgun sequencing, assembly and pathways analysis

For each sample, 3 μg of DNA was placed in the shotgun sequencer at the Novogene Bioinformatics Institute according to the manufacturer's established protocols. Briefly, the DNA was broken into fragments using the Covaris™ S220 System (Applied Biosystems, CA, USA) and extracted using the QIAquick PCR Purification kit (Qiagen, Hilden, Germany). Libraries were prepared following a standard protocol from Illumina, and 100-bp paired-end reads were sequenced using the Illumina HiSeq 2000 platform according to the manufacturer's instructions. The metagenomic datasets are publicly available in the MG-RAST system (http://metagenomics.anl.gov/) under project identifiers from 4543171.3 to 4543182.3.

After quality filtering as described elsewhere^33^, all shotgun metagenomic datasets were rarefied to the same sequencing depth by random resampling 6,144,417,000 bp valid sequences for each sample before downstream analysis. The valid sequences were assembled using SOAPdenovo V 1.06^52^ with the parameters -K 27 (k-mer size)-R-M3-d1. Genes were predicted using MetaGeneMark^53^, and BLASTP^54^ was used to search the protein sequences of the predicted genes within the eggNOG database^55^ and KEGG database^56^ with E ≤ 1 × 10^−5^. The genes were annotated using the eggNOG or KEGG homologs with the lowest e-value. To construct the metabolic pathways associated with the bacterioplankton organisms, sequences were mapped using USEARCH^57^ with E ≤ 1 × 10^−5^ against the KO database. The significant hits were imported into HUMAnN^58^, and the abundance and coverage were calculated for each KEGG metabolic pathway. The abundance data were also analyzed for complete linkage clustering based on the Euclidean distance.

### Statistical analysis

The three datasets generated by 16S rRNA gene sequencing (OTU composition), GeoChip analysis (functional gene signal intensities), and metagenomic shotgun sequencing (relative abundance of KO groups) were further analyzed with one or all of the following statistical methods: (i) α-/β-diversity comparison and response ratio analysis; (ii) clustering based on the community structural or functional characteristics; (iii) dissimilarity test by permutational multivariate analysis of variance (PERMANOVA) with Bray-Curtis dissimilarity for comparing each dataset or sub-dataset; (iv) significance tests based on unpaired Student's *t*-test to identify differences between any two compared objects; (v) CCA and partial CCA to determine the community variation that can be explained by the environmental variables; and (vi) Mantel test to determine the relationship between any two of the targeted matrices. All statistical analyses described above were performed using the R package vegan (R Foundation for Statistical Computing, Vienna, Austria) or our R-based pipeline (http://ieg.ou.edu/microarray/). The analysis of ANOSIM and Raup-Crick were performed by using the software of Past.

## Author Contributions

Q.Y., Y.B., Z.H. and Y.Y. conceived the research. Q.Y., Y.B. and J.L. performed the experiments. Q.Y. wrote the manuscript. Y.B., Y.D., Z.H., J.D.V.N. and J.Z. edited the manuscript. X.W., L.W., Y.D. and Z.S. contributed sampling, reagents or data analysis pipeline. All authors reviewed and accepted the manuscript.

## Supplementary Material

Supplementary Information Supplementary information

## Figures and Tables

**Table 1:** Summary statistics for Mantel tests. The Mantel statistic r(*AB*) estimates the correlation between two proximity matrices, *A* and *B*. Also given is *P*, which can be used to ascertain whether the Mantel regression coefficients were significantly different from zero following 9,999 permutations

Matrix *A*	Matrix *B*	r(*AB*)	*P*
16S rRNA gene sequencing^a^	Environmental factors^d^	−0.524	0.042
GeoChip 5.0^b^	Environmental factors	−0.706	0.002
Metagenomic shotgun sequencing^c^	Environmental factors	−0.564	0.029

^a^Bray-Curtis dissimilarity matrix calculated from the OTU composition classified with a 97% cutoff.

^b^Bray-Curtis dissimilarity matrix calculated from gene signal intensities.

^c^Bray-Curtis dissimilarity matrix calculated from the relative abundance of KEGG orthology.

^d^Euclidean distance matrix calculated from the total phosphorus (TP), ammonium nitrogen (N-NH_4_), nitrate nitrogen (N-NO_3_), chemical oxygen demand (COD), and temperature.

**Table 2:** Summary results from Mantel tests performed at the functional gene level or phylogenetic genus level. The gene/genus numbers and their percentages are given

	*P* < 0.05	*P* ≥ 0.05
Functional genes vs environmental factors	227 (67.0%)	112 (33.0%)
Phylogenetic genera vs environmental factors	19 (11.2%)	150 (88.8%)

**Table 3:** Summary of the major observations that were significantly different between backwater and riverine sites. The detailed data can be found in the supplementary information

Category	Observation	
	Backwater sites > Riverine sites	Backwater sites < Riverine sites
Environmental factors	TN, N-NH_4_ and N-NO_3_	TP, P-PO_4_ and COD
Phytoplankton		Species richness; abundances of *Pyrrophyta* and *Pandorina sp.*
Zooplankton	Abundance of *Daphnia hyaline* and *Sinocalanus dorrii*	Species richness; abundance of *Ciliophora, Trichocerca pusilla, Polyarthra vulgaris, Synchaeta stylata* and *Anuraeopsis fissa*
16S rRNA gene sequencing	Number of detected OTUs; α-diversity; 13 dominant OTUs (mainly *Actinobacteria*); 6 dominant genera; relative abundances of *Actinobacteria, Planctomycetes* and *Verrucomicrobia*	8 dominant OTUs (mainly *Betaproteobacteria*); 2 dominant genera; relative abundances of *Proteobacteria and Gemmatimonadetes*
GeoChip 5.0		Number of detected gene probes; the 74 most abundant functional genes (signal intensities > 100); most of the genes involved in nitrogen cycling
Metagenomic shotgun sequencing	1 and 3 KO groups involved in nitrogen and sulphur metabolisms, respectively	10, 5 and 1 KO groups involved in nitrogen, sulphur and carbon metabolisms, respectively
