# Supplementary material for: Impacts of the Three Gorges Dam on microbial structure and potential function
Source: Sci Rep. 2015 Feb 27;5:8605. doi: 10.1038/srep08605 (PMC4342553; doi:10.1038/srep08605)
Supplement: Supplementary Information [file srep08605-s1.pdf]

Supplementary information

Impacts of the Three Gorges Dam on microbial structure and potential function

Qingyun Yan[1,2]*, Yonghong Bi[1]*, Ye Deng[2,5], Zhili He[2], Liyou Wu[2], Joy D. Van Nostrand[2], Zhou Shi[2], Jinjin Li[1,6], Xi Wang[1,6], Zhengyu Hu[1], Yuhe Yu[1] & Jizhong Zhou[2,3,4]

[1]State Key Laboratory of Freshwater Ecology and Biotechnology, Institute of Hydrobiology, Chinese Academy of Sciences, Wuhan, China

[2]Institute for Environmental Genomics and Department of Microbiology and Plant Biology, University of Oklahoma, Norman, OK, USA

[3]State Key Joint Laboratory of Environment Simulation and Pollution Control, School of Environment, Tsinghua University, Beijing, China

[4]Earth Sciences Division, Lawrence Berkeley National Laboratory, Berkeley, CA, USA

[5]CAS Key Laboratory of Environmental Biotechnology, Research Center for Eco-Environmental Sciences, Chinese Academy of Sciences, Beijing, China

[6]Graduate University of Chinese Academy of Sciences, Beijing, 100049, China

*These authors contributed equally to this work.

Correspondence and requests for materials should be addressed to Y.Y. (yhyu@ihb.ac.cn) or J.Z. (jzhou@ou.edu).

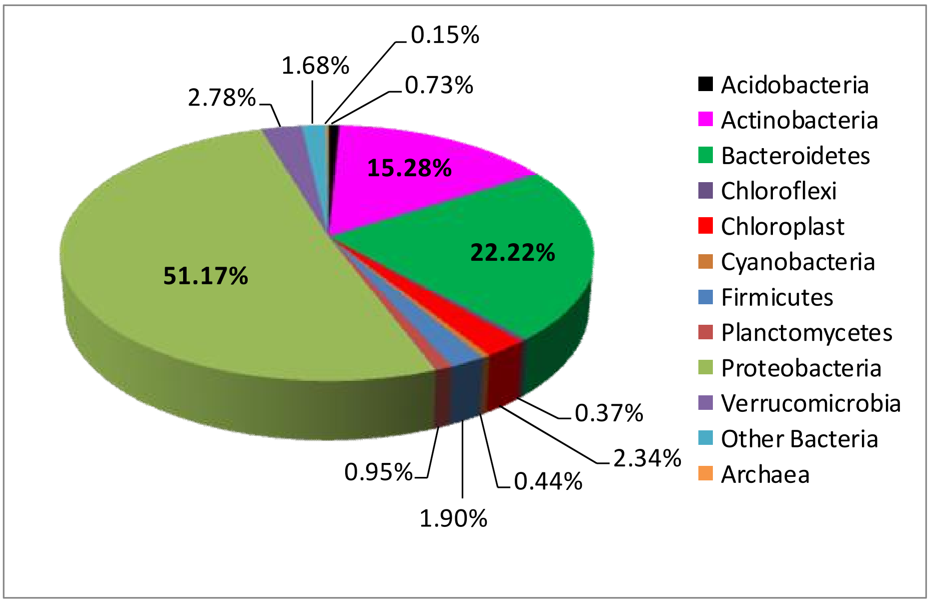

Figure S1 The percentages of OTUs assigned to major bacterial phyla (97% cutoff).

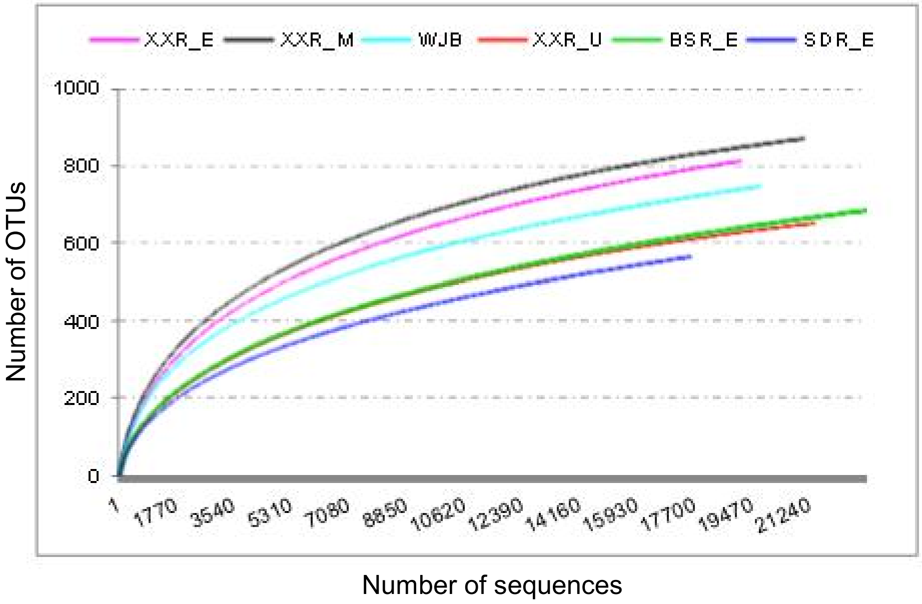

Figure S2 Rarefaction curves showing the observed OTU richness (97%) with increasing sequencing depth. The estuary, midstream and upstream sites along the Xiangxi River are abbreviated as XXR_E, XXR_M, and XXR_U, respectively; the other three sites are the Wujia Bay (WJB), the estuary of the Baisha River (BSR_E) and the estuary of the Shendu River (SDR_E).

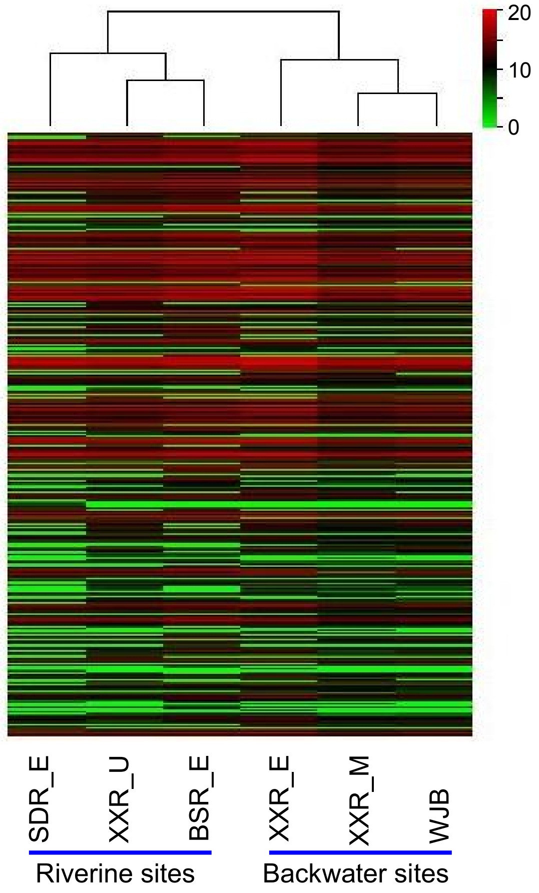

Figure S3 Heatmap showing the general differences among the six investigated communities based on all detected KEGG ortholog groups. The full name of each sampling site is given in Figure S2.

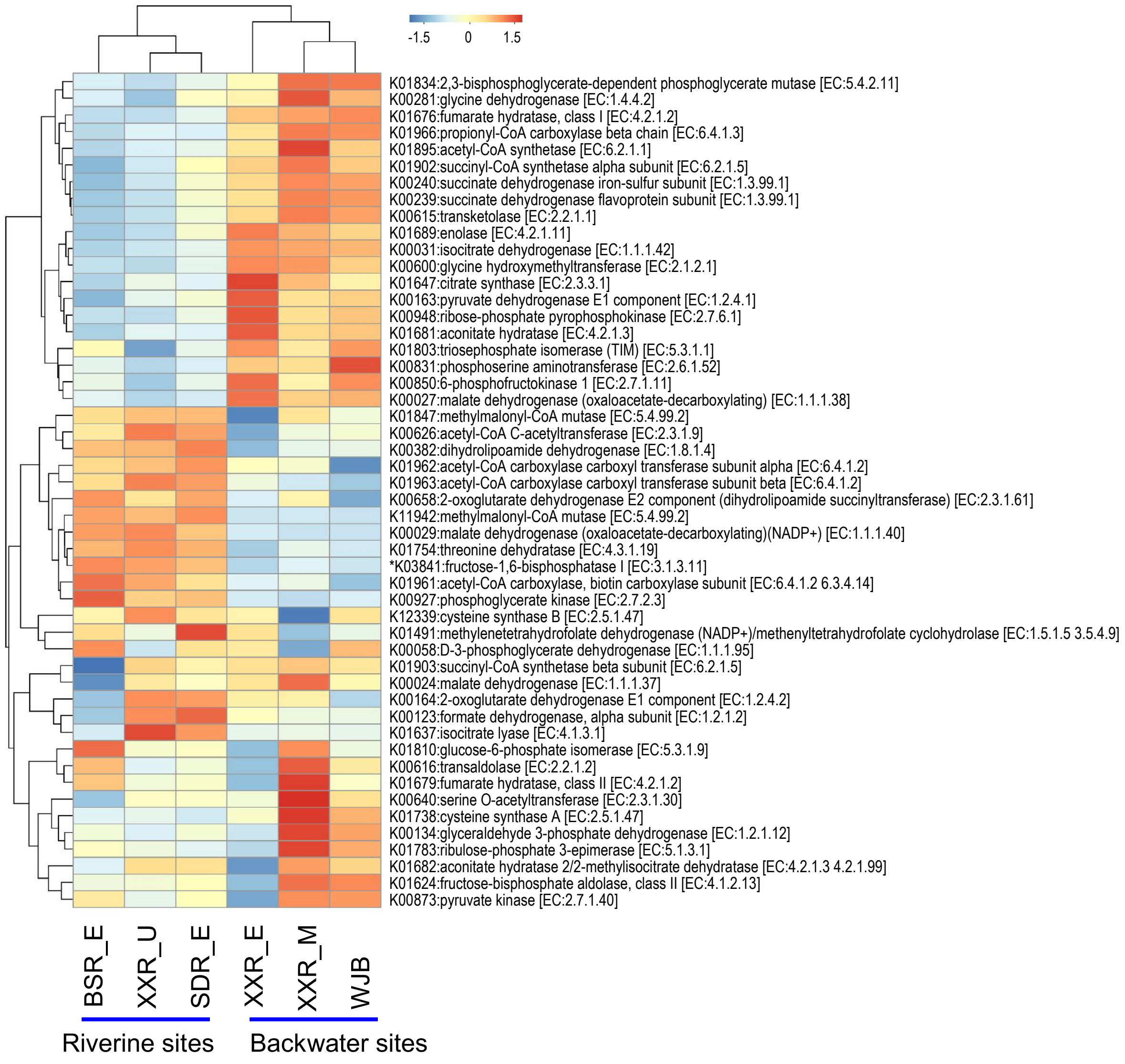

Figure S4 Heatmap showing the differences among the six investigated communities based on the KEGG orthology groups involved in carbon metabolism pathways (ko01200). Only the 50 most abundant groups are included, an asterisk indicates there was significant difference between backwater and riverine sites determined by response ratio analysis at a 95% confidence interval. The full name of each sampling site is listed in Figure S2.

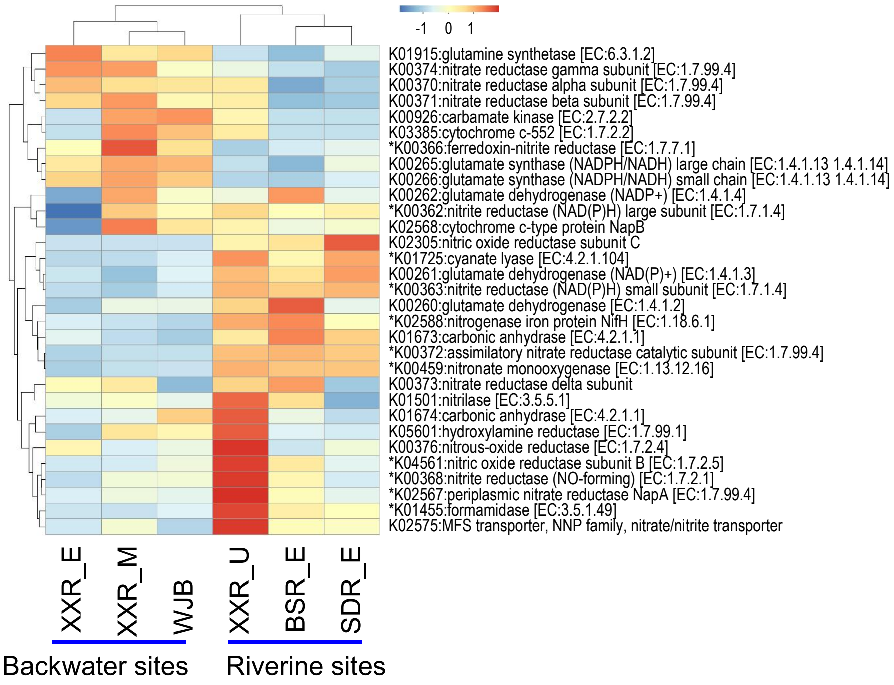

Figure S5 Heatmap showing the differences among the six investigated communities based on the KEGG orthology groups involved in nitrogen metabolism pathways (ko00910). An asterisk indicates there was a significant difference between backwater and riverine sites determined by response ratio analysis at a 95% confidence interval. The full name of each sampling site is listed in Figure S2.

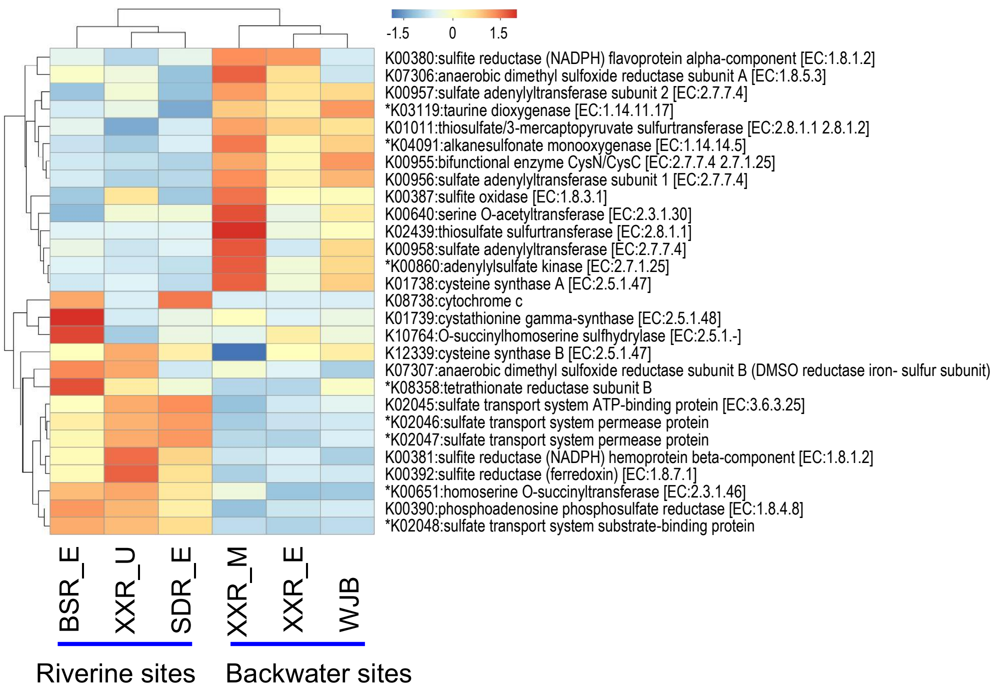

Figure S6 Heatmap showing the differences among the six investigated communities based on the KEGG orthology groups involved in sulphur metabolism pathways (ko00920). An asterisk indicates there was a significant difference between backwater and riverine sites determined by response ratio analysis at a 95% confidence interval. The full name of each sampling site is listed in Figure S2.

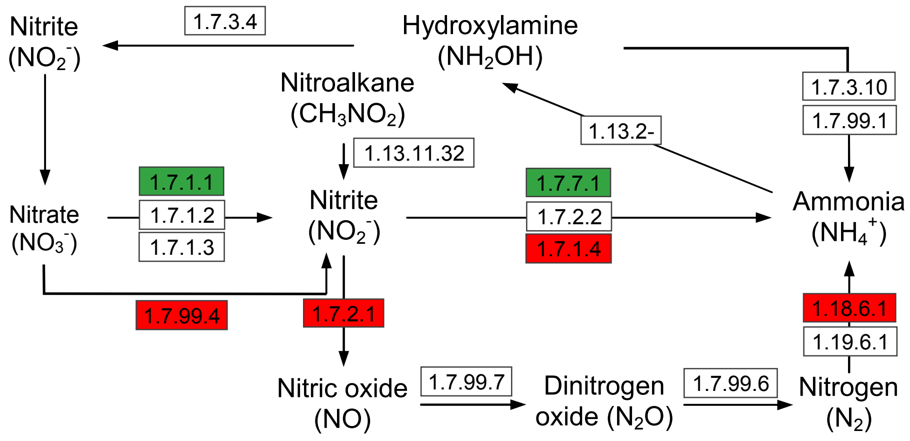

Figure S7 Identified enzymes involved in nitrogen metabolism pathways. Red-coloured frames indicate the enzymes were significantly lower in backwater sites compared to riverine sites, green-coloured frames indicate enzymes that were significantly higher in backwater sites (determined by response ratio analysis ata 95% confidence interval), white frames indicate no significant difference were observed. The names of enzymes corresponding to each enzyme code is listed in Figure S5.

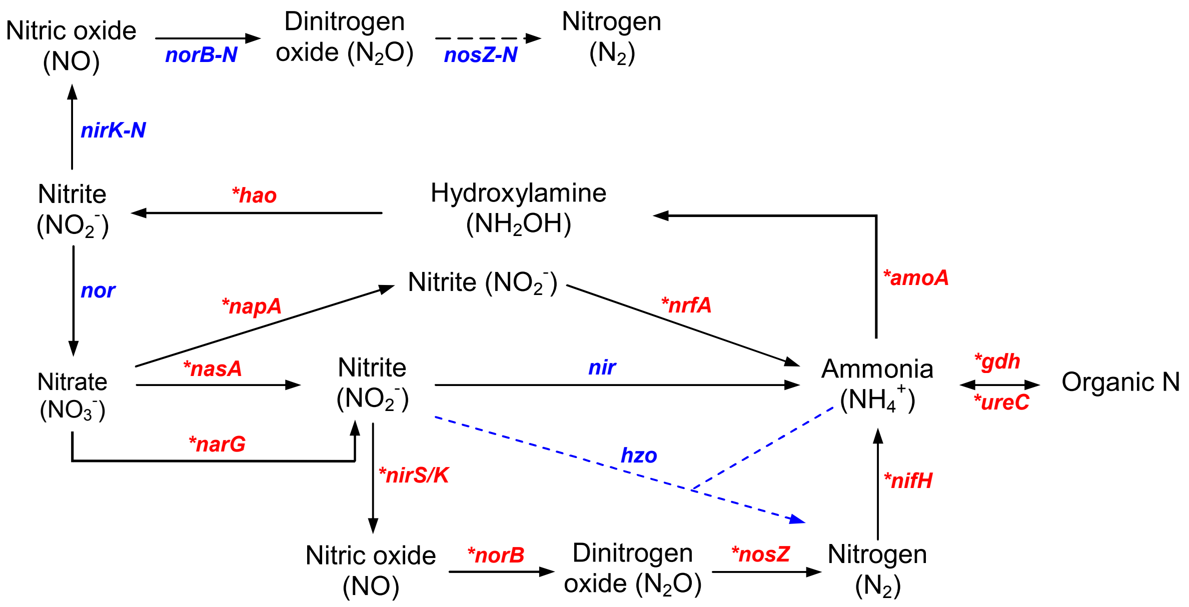

Figure S8 Comparison of GeoChip signal intensities for genes involved in nitrogen cycling. Red-coloured genes with asterisks indicate significantly higher signal intensities in riverine samples compared to backwater samples (determined by response ratio analysis at the significant level of 95% confidence interval); blue-coloured genes were not present on GeoChip 5.0, or were undetected in those samples.

Table S1 Summary of sample physicochemical characteristics.

Sample ID	TP[a] (mg L^{-1})	P-PO_4[a] (mg L^{-1})	COD[a] (mg L^{-1})	N-NH_4[b] (μg mL^{-1})	N-NO_3[b] (μg mL^{-1})	TN[b] (μg mL^{-1})	Chl *a* (μg L^{-1})	Turb (NTU)	Tran (m)	AT (°C)	WT (°C)	DO (mg L^{-1})	Cond (mS cm^{-1})	pH	ORP (mV)
XXR_E	0.20	0.16	1.13	0.12	1.54	1.77	4.63	1.30	5.10	20.0	17.4	7.96	374.9	8.24	255.0
XXR_M	0.24	0.14	1.94	0.14	0.80	1.24	30.50	5.80	0.90	26.5	18.7	12.26	332.3	8.83	211.7
WJB	0.14	0.01	2.59	0.04	0.23	0.89	67.12	6.40	0.70	27.0	19.7	19.43	266.8	9.28	209.0
XXR_U	0.45	0.31	3.07	0.03	0.08	0.71	56.62	3.80	1.40	31.0	23.3	16.42	282.5	9.52	185.1
BSR_E	0.72	0.51	2.99	0.03	0.07	0.83	82.80	11.60	0.50	30.0	20.8	15.01	266.1	9.25	199.4
SDR_E	0.54	0.35	3.56	0.06	0.02	0.99	89.61	11.60	0.60	30.0	20.2	16.43	271.4	9.24	178.2

[a]Concentrations in backwater sites (XXR_E, XXR_M, WJB) were significantly lower than those in riverine sites (XXR_U, BSR_E, SDR_E), [b]Concentrations in backwater sites are significantly higher than those in riverine sites. Significance determined by response ratio analysis at a 95% confidence interval.

Abbreviations: Chl *a*, Chlorophyll a; TP, total phosphorus; P-PO_4, phosphate phosphorus; N-NH_4, ammonium nitrogen; N-NO_3, nitrate nitrogen; TN, total nitrogen; COD, chemical oxygen demand; Turb, Turbidity; Tran, transparency; AT, air temperature; WT, water temperature; DO, dissolved oxygen; Cond, conductivity; ORP, oxidation-reduction potential. The full name of each sampling site is listed in Figure S2.

Table S2 Abundance of phyto- and zooplankton taxa determined morphologically (given are mean value and standard deviation, SD)

(Red indicate significant differences between backwater and riverine sites)

Group	Taxa	Backwater sites		Riverine sites		*P* (t-test)
		Mean	SD	Mean	SD	
**Algae** (10^4 ind. L^{-1})	*Cryptomonas* sp.	432.7	363	239.1	31.6	0.409
	Pyrrophyta			7.8	4.4	**0.038**
	Euglena sp.			3.6	3.3	0.132
	Melosira sp.	17.5	19	27	19.1	0.573
	Cyclotella sp.	13257.6	12416.7	2898.9	819	0.223
	Navicula sp.			1.4	1.2	0.116
	Synedra sp.	17.1	29.6	26.3	9.9	0.634
	Pinnularia sp.			0.7	1.2	0.374
	Cocconeis sp.			0.7	1.2	0.374
	Diatoma sp.			1.4	2.5	0.374
	Gomphonema sp.			1.4	2.5	0.374
	Cymbella sp.			0.7	1.2	0.374
	Achnanthes sp.			0.7	1.2	0.374
	Asterionella sp.			1.4	2.5	0.374
	Surirella sp.			1.4	2.5	0.374
	Scenedesmus sp.			7.1	6.5	0.132
	Chlamydomonas sp.	7.9	6.9	10.7	11.3	0.736
	Chodatella sp.	4.3	7.4	1.4	2.5	0.561
	Ankistrodesmus sp.			0.7	1.2	0.374
	Pandorina sp.			552.2	229.3	**0.014**
	Chlorococcales			8.5	8.5	0.158
**Protozoa** (ind. L^{-1})	*Epistylis* sp.	5000	6032.4	300	519.6	0.25
	Strobilidium sp.	5400	9094.5			0.362
	Didinium balbianii nanum			2500	2042.1	0.101
	Tintinnopsis sinensis			7800	6285.7	0.098
	Phascolodon vorticella			300	519.6	0.374
	Chilodonella cucullus			200	173.2	0.116
	Trachelophyllum chilense			100	173.2	0.374
	Hypotricha			200	173.2	0.116
	Unidentified *Ciliophora*	200	346.4	4200	1587.5	**0.013**
**Rotifers** (ind. L^{-1})	*Conochilus* sp.	460	398.4			0.116
	Keratella cochlearis			3690	6158.9	0.358
	Trichocerca pusilla			7110	1866.5	**0.003**
	Polyarthra vulgaris			890	391.5	**0.017**
	Synchaeta stylata			210	30	**0**
	Keratella quadrata			20	34.6	0.374
	Anuraeopsis fissa			130	75.5	**0.041**
	Filinia longiseta			50	62.5	0.238
	Unidentified *Rotaria*			60	104	0.374
**Cladocera** (ind. L^{-1})	*Daphnia hyaline*	7.7	2.1	0.1	0.1	**0.003**
	Bosmina longirostris	54.3	50.6	4.2	4.3	0.162
	Ilyocryptus sp.	1	1.7	0.2	0.3	0.457
	Bosminopsis deitersi	1	1	0.4	0.7	0.56
	Alona intermedia			0.08	0.14	0.374
**Copepods** (ind. L^{-1})	*Cyclops* sp.			0.08	0.14	0.374
	Sinocalanus dorrii	5.83	2.53			**0.016**
	Schmackeria forbesi	0.08	0.14			0.374

Table S3.1 Abundance of the dominant genura (relative abundance > 1%, given are the mean value and standard deviation, SD) determined by 16S rRNA gene sequencing

(Red indicate significant differences between backwater and riverine sites)

Phylum	Class	Order	Family	Genus	Backwater sites		Riverine sites		*P* (t-test)
					Mean	SD	Mean	SD	
Actinobacteria	*Actinobacteria*	*Acidimicrobiales*	*Acidimicrobiaceae*	*Ilumatobacter*	0.0111	0.0027	0.0000	0.0000	**0.012**
Bacteroidetes	*Flavobacteria*	*Flavobacteriales*	*Cryomorphaceae*	*Lishizhenia*	0.0180	0.0034	0.0012	0.0004	**0.001**
	Flavobacteria	*Flavobacteriales*	*Flavobacteriaceae*	*Flavobacterium*	0.0135	0.0027	0.0845	0.0324	**0.019**
	Sphingobacteria	*Sphingobacteriale*	*Chitinophagaceae*	*Sediminibacterium*	0.0825	0.0136	0.1125	0.0416	0.301
	Sphingobacteria	*Sphingobacteriale*	*Cyclobacteriaceae*	*Algoriphagus*	0.0273	0.0141	0.0026	0.0013	**0.04**
	Sphingobacteria	*Sphingobacteriale*	*Cytophagaceae*	*Arcicella*	0.0151	0.0040	0.0295	0.0126	0.134
Cyanobacteria/	*Chloroplast*	Unclassified	*Chloroplast*	*Bacillariophyta*	0.0800	0.0885	0.0128	0.0096	0.261
Chloroplast	*Chloroplast*	Unclassified	*Chloroplast*	*Cryptomonadacea*	0.0055	0.0077	0.0009	0.0009	0.36
Proteobacteria	*Alphaproteobacteria*	*Sphingomonadale*	*Sphingomonadacea*	*Sandarakinorhabd*	0.0062	0.0036	0.0001	0.0001	1
	Alphaproteobacteria	*Sphingomonadale*	*Sphingomonadacea*	*Sphingomonas*	0.0176	0.0059	0.0047	0.0012	**0.02**
	Betaproteobacteria	*Burkholderiales*	*Burkholderiaceae*	*Polynucleobacter*	0.0259	0.0049	0.0086	0.0019	**0.005**
	Betaproteobacteria	*Burkholderiales*	*Burkholderiales_inc*	*Aquabacterium*	0.0013	0.0004	0.0067	0.0039	0.075
	Betaproteobacteria	*Burkholderiales*	*Comamonadaceae*	*Acidovorax*	0.0125	0.0037	0.0236	0.0069	0.07
	Betaproteobacteria	*Burkholderiales*	*Comamonadaceae*	*Limnohabitans*	0.0085	0.0023	0.0296	0.0015	**0**
	Betaproteobacteria	*Burkholderiales*	*Comamonadaceae*	*Polaromonas*	0.0077	0.0000	0.0172	0.0109	0.208
	Betaproteobacteria	*Rhodocyclales*	*Rhodocyclaceae*	*Thauera*	0.0072	0.0056	0.0002	0.0003	0.196
	Gammaproteobacter	*Xanthomonadales*	*Xanthomonadacea*	*Arenimonas*	0.0080	0.0035	0.0005	0.0004	**0.022**
Verrucomicrobi	*Opitutae*	*Puniceicoccales*	*Puniceicoccaceae*	*Cerasicoccus*	0.0123	0.0072	0.0010	0.0007	0.054

Table S3.2 Abundance of the dominant OTUs (relative abundance > 1%, given are the mean value and standard deviation, SD) determined by 16S rRNA gene sequencing

(Red indicate significant differences between backwater and riverine sites)

OTUs	Phylum	Class	Order	Family	Genus	Backwater sites		Riverine sites		*P* (t-test)
						Mean	SD	Mean	SD	
OTU_1	***Actinobacteria***	*Actinobacteria*	*Acidimicrobiales*	*Acidimicrobiacea*	*Ilumatobacter*	0.0063	0.0024	0.0000	0.0000	1
OTU_2		*Actinobacteria*	*Actinomycetales*	*Nakamurellacea*	Unclassified	0.0828	0.0548	0.0449	0.0080	0.302
OTU_3		*Actinobacteria*	*Actinomycetales*	Unclassified	Unclassified	0.0068	0.0042	0.0015	0.0007	0.094
OTU_4		*Actinobacteria*	*Actinomycetales*	Unclassified	Unclassified	0.0200	0.0084	0.0020	0.0003	**0.02**
OTU_5		*Actinobacteria*	*Actinomycetales*	Unclassified	Unclassified	0.0089	0.0034	0.0001	0.0001	**0.011**
OTU_6		*Actinobacteria*	*Actinomycetales*	Unclassified	Unclassified	0.0065	0.0025	0.0002	0.0001	**0.012**
OTU_7		*Actinobacteria*	*Actinomycetales*	Unclassified	Unclassified	0.0144	0.0018	0.0002	0.0001	**0**
OTU_8		*Actinobacteria*	*Actinomycetales*	Unclassified	Unclassified	0.0282	0.0127	0.0064	0.0025	**0.043**
OTU_9		*Actinobacteria*	*Actinomycetales*	Unclassified	Unclassified	0.0144	0.0038	0.0001	0.0000	**0.003**
OTU_10		*Actinobacteria*	*Actinomycetales*	Unclassified	Unclassified	0.0060	0.0013	0.0001	0.0000	**0.002**
OTU_11	***Bacteroidetes***	*Flavobacteria*	*Flavobacteriales*	*Cryomorphacea*	*Lishizhenia*	0.0090	0.0031	0.0006	0.0004	**0.01**
OTU_12		*Flavobacteria*	*Flavobacteriales*	*Cryomorphacea*	Unclassified	0.0048	0.0051	0.0001	0.0002	1
OTU_13		*Flavobacteria*	*Flavobacteriales*	*Flavobacteriacea*	*Flavobacterium*	0.0015	0.0002	0.0107	0.0094	0.164
OTU_14		*Flavobacteria*	*Flavobacteriales*	*Flavobacteriacea*	*Flavobacterium*	0.0008	0.0007	0.0153	0.0046	**0.006**
OTU_15		*Flavobacteria*	*Flavobacteriales*	*Flavobacteriacea*	*Flavobacterium*	0.0046	0.0027	0.0350	0.0102	**0.007**
OTU_16		*Sphingobacteria*	*Sphingobacteriale*	*Chitinophagacea*	*Sediminibacter*	0.0504	0.0090	0.0806	0.0268	0.138
OTU_17		*Sphingobacteria*	*Sphingobacteriale*	*Chitinophagacea*	*Sediminibacter*	0.0199	0.0033	0.0176	0.0086	0.697
OTU_18		*Sphingobacteria*	*Sphingobacteriale*	*Cyclobacteriacea*	*Algoriphagus*	0.0239	0.0126	0.0023	0.0010	**0.042**
OTU_19		*Sphingobacteria*	*Sphingobacteriale*	*Cytophagaceae*	*Arcicella*	0.0151	0.0040	0.0294	0.0126	0.134
OTU_20		*Sphingobacteria*	*Sphingobacteriale*	Unclassified	Unclassified	0.0067	0.0041	0.0005	0.0001	0.059
OTU_21		*Sphingobacteria*	*Sphingobacteriale*	Unclassified	Unclassified	0.0002	0.0002	0.0197	0.0093	**0.037**
OTU_22		*Sphingobacteria*	*Sphingobacteriale*	Unclassified	Unclassified	0.0227	0.0142	0.0008	0.0004	0.056
OTU_23	***Cyanobacteria/***	*Chloroplast*	Unclassified	*Chloroplast*	*Bacillariophyt*	0.0123	0.0138	0.0020	0.0019	0.269
OTU_24	***Chloroplast***	*Chloroplast*	Unclassified	*Chloroplast*	*Bacillariophyt*	0.0608	0.0666	0.0098	0.0075	0.258
OTU_25		*Chloroplast*	Unclassified	*Chloroplast*	*Cryptomonad*	0.0043	0.0059	0.0001	0.0001	0.311

Table S3.2 (Cont.)

OTUs	Phylum	Class	Order	Family	Genus	Backwater sites		Riverine sites		*P* (t-test)
						Mean	SD	Mean	SD	
OTU_26	***Proteobacteria***	*Alphaproteobact*	*Sphingomonadale*	*Sphingomonada*	*Sphingomonas*	0.0098	0.0038	0.0025	0.0009	**0.03**
OTU_27		*Betaproteobacte*	*Burkholderiales*	*Burkholderiacea*	*Polynucleobac*	0.0191	0.0036	0.0058	0.0009	**0.003**
OTU_28		*Betaproteobacte*	*Burkholderiales*	*Burkholderiales_*	Unclassified	0.0301	0.0106	0.0426	0.0050	0.139
OTU_29		*Betaproteobacte*	*Burkholderiales*	*Comamonadace*	*Acidovorax*	0.0107	0.0043	0.0217	0.0068	0.077
OTU_30		*Betaproteobacte*	*Burkholderiales*	*Comamonadace*	*Limnohabitans*	0.0013	0.0003	0.0108	0.0005	**0**
OTU_31		*Betaproteobacte*	*Burkholderiales*	*Comamonadace*	*Limnohabitans*	0.0044	0.0013	0.0092	0.0015	**0.015**
OTU_32		*Betaproteobacte*	*Burkholderiales*	*Comamonadace*	*Polaromonas*	0.0074	0.0003	0.0165	0.0106	0.211
OTU_33		*Betaproteobacte*	*Burkholderiales*	*Comamonadace*	Unclassified	0.0065	0.0033	0.0330	0.0051	**0.002**
OTU_34		*Betaproteobacte*	*Burkholderiales*	*Comamonadace*	Unclassified	0.0020	0.0008	0.0162	0.0040	**0.004**
OTU_35		*Betaproteobacte*	*Burkholderiales*	*Comamonadace*	Unclassified	0.0392	0.0201	0.2996	0.0936	**0.009**
OTU_36		*Betaproteobacte*	*Burkholderiales*	Unclassified	Unclassified	0.0411	0.0177	0.0007	0.0001	**0.017**
OTU_37		*Betaproteobacte*	*Rhodocyclales*	*Rhodocyclaceae*	*Thauera*	0.0067	0.0052	0.0002	0.0002	0.194
OTU_38		*Gammaproteoba*	*Xanthomonadales*	*Xanthomonadac*	*Arenimonas*	0.0061	0.0028	0.0004	0.0004	**0.024**
OTU_39		Unclassified	Unclassified	Unclassified	Unclassified	0.0121	0.0090	0.0003	0.0002	0.086
OTU_40	***Verrucomicrobia***	*Opitutae*	*Puniceicoccales*	*Puniceicoccacea*	*Cerasicoccus*	0.0097	0.0058	0.0010	0.0007	0.063
OTU_41	**Unclassified**	Unclassified	Unclassified	Unclassified	Unclassified	0.0069	0.0052	0.0000	0.0000	1

Table S4 Relative abundance of the major bacterial and archaeal taxa determined by 16S rRNA gene sequencing. Numbers in cells are percentages of relative abundance, highest abundances are shown, middle values in blue and lowest values in white. An asterisk indicates a significant different between backwater and riverine sites (*t*-test, $P < 0.05$).

Taxonomy	Backwater sites			Riverine sites		
	XXR_E	XXR_M	WJB	XXR_U	BSR_E	SDR_E
Acidobacteria	0.08	0.46	0.01	0.02	0.03	0.06
Actinobacteria*	30.93	18.78	37.45	6.18	6.00	8.58
Bacteroidetes	29.17	25.59	24.09	17.27	34.11	32.51
Incertae sedis	0.17	0.07	0.09	0.10	0.11	0.13
Bacteroidia	0.17	1.20	0.93	0.09	0.17	0.14
Flavobacteria	6.20	7.57	5.00	6.14	12.48	10.22
Sphingobacteria	22.64	16.75	18.07	10.95	21.35	22.03
Chloroplast	4.49	18.60	3.07	0.82	2.70	1.13
Cyanobacteria	0.04	0.07	0.07	0.01	0.05	0.00
Firmicutes	0.10	0.23	0.06	0.15	0.13	0.34
Fusobacteria	0.03	0.03	0.02	0.07	0.03	0.05
Gemmatimonadetes*	0.02	0.03	0.03	0.16	0.07	0.10
Planctomycetes*	0.09	0.31	0.22	0.01	0.02	0.02
Proteobacteria*	32.93	33.20	34.08	74.67	56.48	56.89
Alphaproteobacteria	2.62	6.03	6.31	3.51	3.88	3.01
*Betaproteobacteria**	27.43	21.88	25.15	70.23	51.63	53.27
Deltaproteobacteria	1.02	2.25	0.47	0.43	0.38	0.26
Epsilonproteobacteria	0.00	0.05	0.01	0.04	0.03	0.01
*Gammaproteobacteria**	1.85	2.98	2.14	0.46	0.57	0.34
Verrucomicrobia*	2.04	2.43	0.81	0.46	0.33	0.28
Opitutae	1.83	1.90	0.44	0.05	0.18	0.12
Spartobacteria	0.11	0.19	0.14	0.25	0.06	0.06
Sub division 3	0.06	0.05	0.01	0.02	0.01	0.00
Verrucomicrobiae	0.04	0.30	0.22	0.14	0.09	0.10
**Other bacterial**	0.07	0.19	0.07	0.17	0.04	0.05
**Archaea**	0.01	0.07	0.02	0.01	0.00	0.01

The full name of each sampling site is listed in Figure S2.

Table S5 Normalized signal intensities (given are the mean value and standard deviation, SD) of the most abundant functional genes (signal intensities > 100) determined by GeoChip 5.0

(Red indicate significant differences between backwater and riverine sites)

Gene category	Subcategory	gene	Backwater sites		Riverine sites		*P* (t-test)
			Mean	SD	Mean	SD	
**Carbon cycling**	Carbon degradation	*acetylglucosaminidase*	279.21	10.51	358.82	11.51	**0.001**
	Carbon degradation	*alginase*	81.15	1.80	99.27	2.82	**0.001**
	Carbon degradation	*amyA*	1803.57	83.48	2223.66	57.72	**0.002**
	Carbon degradation	*ara*	268.64	12.85	331.20	12.21	**0.004**
	Carbon degradation	*cda*	88.78	2.86	109.42	3.26	**0.001**
	Carbon degradation	*cellobiase*	190.46	5.98	249.94	7.80	**0.001**
	Carbon degradation	*chitinase*	402.17	12.68	503.83	13.16	**0.001**
	Carbon degradation	*cutinase*	167.40	6.75	201.11	5.42	**0.003**
	Carbon degradation	*endochitinase*	134.04	5.72	173.53	3.17	**0.001**
	Carbon degradation	*endoglucanase*	114.83	3.02	142.05	3.71	**0.001**
	Carbon degradation	*glucoamylase*	83.76	6.23	101.29	5.13	**0.02**
	Carbon degradation	*mannanase*	122.87	4.83	150.56	3.99	**0.002**
	Carbon degradation	*pectinase*	87.19	3.94	110.61	2.79	**0.01**
	Carbon degradation	*phenol oxidase*	185.49	5.37	241.09	12.35	**0.002**
	Carbon degradation	*pme*	99.03	1.56	129.06	7.09	**0.002**
	Carbon degradation	*RgaE*	112.91	5.05	142.76	6.20	**0.003**
	Carbon degradation	*rgl*	105.99	2.53	122.59	1.18	**0.001**
	Carbon degradation	*vana*	104.18	3.86	131.10	2.46	**0.001**
	Carbon degradation	*xyla*	148.11	4.70	185.70	6.09	**0.001**
	Carbon degradation	*xylanase*	232.34	15.02	298.79	10.59	**0.003**
	Carbon fixation	*ccmL*	93.64	3.62	125.42	2.38	**0**
	Carbon fixation	*CsoS1 CcmK*	170.92	7.48	226.07	5.01	**0**
	Carbon fixation	*FBP aldolase*	73.85	6.87	100.11	3.98	**0.005**
	Carbon fixation	*FBPase*	162.39	5.83	218.90	6.22	**0**
	Carbon fixation	*fthfs*	247.04	14.92	319.82	12.89	**0.003**
	Carbon fixation	*GAPDH Calvin*	151.32	6.25	199.83	6.42	**0.001**

Table S5 (Cont.)

Gene category	Subcategory	gene	Backwater sites		Riverine sites		*P* (t-test)
			Mean	SD	Mean	SD	
**Carbon cycling**	Carbon fixation	*pgk*	95.56	5.21	121.22	5.49	**0.004**
	Carbon fixation	*PRI*	106.79	8.18	147.82	5.02	**0.002**
	Carbon fixation	*rubisco*	156.13	4.12	200.19	5.82	**0**
	Carbon fixation	*TIM*	146.29	10.20	178.95	6.15	**0.009**
	Carbon fixation	*tktA*	284.91	13.48	363.12	11.99	**0.002**
	Methane	*mcra*	76.37	3.60	99.13	2.55	**0.001**
**Metal homeostasis**	Arsenic	*aoxb*	103.43	9.68	132.90	3.11	**0.007**
	Arsenic	*arsc*	337.85	25.36	430.22	19.26	**0.007**
	Mercury	*mer*	293.68	10.00	362.91	7.58	**0.001**
	Tellurium	*tehb*	75.00	3.37	105.10	4.64	**0.001**
	Tellurium	*terc*	325.77	9.47	411.19	10.71	**0.001**
**Nitrogen cycling**	Ammonification	*gdh*	103.54	7.04	127.42	5.47	**0.01**
	Ammonification	*urec*	260.03	14.80	324.17	10.65	**0.004**
	Denitrification	*narg*	449.57	18.66	568.70	13.15	**0.001**
	Denitrification	*nirk*	172.78	8.80	237.00	10.79	**0.001**
	Denitrification	*nirs*	196.34	8.00	270.00	9.64	**0.001**
	Denitrification	*nosz*	255.98	13.42	346.68	10.82	**0.001**
	Nitrogen fixation	*nifh*	363.76	17.86	474.01	14.91	**0.001**
**Organic remediation**	Aromatics	*bphF1*	114.07	7.69	145.96	4.42	**0.003**
	Aromatics	*one ring 23diox*	102.03	3.32	135.90	3.13	**0**
	Aromatics	*arylest*	95.35	4.56	124.36	7.44	**0.005**
	Aromatics	*badh*	104.07	4.68	132.28	6.00	**0.003**
	Aromatics	*catechol*	124.58	6.00	152.89	3.78	**0.002**
	Aromatics	*catechol b*	105.59	7.90	133.88	3.41	**0.005**
	Aromatics	*cmci*	84.74	3.80	113.75	2.80	**0**
	Aromatics	*mdlc*	112.47	4.57	131.98	3.51	**0.004**
	Aromatics	*pcag*	108.14	5.40	137.13	3.99	**0.002**
	Aromatics	*poba*	150.82	4.57	187.52	4.40	**0.001**

Table S5 (Cont.)

Gene category	Subcategory	gene	Backwater sites		Riverine sites		*P* (t-test)
			Mean	SD	Mean	SD	
**Organic remediation**	Aromatics	*tfda*	71.10	3.70	100.77	3.17	**0.001**
	Aromatics	*xylj*	92.68	1.74	104.81	2.74	**0.003**
	Chlorinated solvents	*dehh109*	92.09	5.19	124.61	3.97	**0.001**
	Chlorinated solvents	*exaa (moxf)*	87.61	1.42	107.80	3.23	**0.001**
	Herbicides related compound	*pcpe*	88.78	6.47	119.83	2.64	**0.002**
	Herbicides related compound	*phn*	135.98	5.63	174.41	4.53	**0.001**
	Other hydrocarbons	*alkb*	176.32	10.65	223.71	6.79	**0.003**
	Other hydrocarbons	*chnb*	81.45	1.74	107.75	3.62	**0**
	Pesticides related compound	*linb*	130.91	5.08	161.65	3.76	**0.001**
**Phosphorus cycling**	Phytic acid hydrolysis	*phytase*	95.25	2.62	119.37	1.26	**0**
	Polyphosphate degradation	*ppx*	512.94	22.28	654.54	18.96	**0.001**
	Polyphosphate synthesis	*ppk*	181.08	10.96	228.73	8.71	**0.004**
**Sulphur cycling**	adenylylsulfate reductase	*aps apra*	93.35	5.83	113.72	4.20	**0.008**
	Other	*cysI*	117.75	7.03	140.40	3.79	**0.008**
	Reduction	*cysJ*	186.12	7.95	231.38	4.39	**0.001**
	sulfite reduction	*dsra*	341.70	24.42	455.28	18.88	**0.003**
	sulfite reduction	*dsrB*	266.94	12.03	339.52	11.74	**0.002**
	sulfite reduction	*Sir*	90.23	3.03	113.53	4.86	**0.002**
	Sulfur oxidation	*soxY*	110.76	5.26	137.12	2.00	**0.001**
**Virulence**	Antibiotic resistance	*B lactamase A*	92.82	4.83	114.93	1.07	**0.002**

Table S6 Normalized signal intensities of functional genes detected by GeoChip 5.0. Numbers in cells are normalized signal intensities of the detected genes, highest values shown in red, middle values in blue and lowest values in white. An asterisk indicates a significant difference between backwater and riverine sites (*t*-test, $P < 0.05$).

	Backwater sites			Riverine sites		
	XXR_E	XXR_M	WJB	XXR_U	BSR_E	SDR_E
**Carbon cycling***	**7454.40**	**7959.10**	**8120.11**	**9596.38**	**10190.14**	**9945.02**
Carbon degradation*	5458.18	5813.48	5921.24	6939.55	7355.87	7185.65
Carbon fixation*	1875.19	2020.88	2063.48	2493.13	2659.65	2593.06
Methane*	121.03	124.74	135.39	163.70	174.62	166.31
**Metal homeostasis***	**1244.01**	**1348.41**	**1364.58**	**1617.03**	**1715.04**	**1673.18**
Arsenic*	472.36	531.79	543.87	629.74	678.32	665.41
Chromium*	18.30	20.82	20.97	24.64	25.51	25.47
Copper*	19.22	19.02	20.20	21.75	22.91	22.63
Mercury*	343.85	364.50	362.06	426.37	444.43	432.13
Silicon*	3.73	7.38	6.65	13.38	12.74	10.99
Tellurium*	386.55	404.90	410.83	501.16	531.13	516.55
**Nitrogen***	**2134.40**	**2286.14**	**2354.34**	**2821.20**	**3004.64**	**2931.50**
Ammonification*	339.60	370.09	381.01	435.64	467.54	451.58
Anammox*	10.27	9.54	9.33	10.73	11.62	11.58
Assimilation	3.30	4.05	4.14	4.11	5.02	5.18
Assimilatory N reduction*	164.14	185.56	190.73	212.17	228.36	226.02
Denitrification*	1074.49	1131.40	1171.40	1439.25	1529.88	1491.37
Dissimilatory N reduction*	110.87	117.71	128.61	148.40	157.29	158.69
N assimilation*	49.71	50.08	53.56	62.28	63.31	63.23
Nitrification*	38.87	43.21	41.91	48.76	52.06	51.24
Nitrogen fixation*	343.15	374.49	373.65	459.85	489.57	472.61
**Organic remediation***	**3730.09**	**3951.43**	**4076.38**	**4850.82**	**5128.89**	**5011.67**
Aromatics*	398.90	421.35	433.04	519.95	547.74	532.98
Chlorinated solvents*	0.98	0.92	1.02	1.11	1.11	1.12
Polycyclic aromatics*	5.85	6.02	5.04	8.68	9.54	9.47
Aromatics*	2009.89	2121.54	2191.24	2565.83	2717.83	2665.72
Chlorinated solvents*	247.30	266.20	275.83	344.89	363.59	345.67
Halogenated compounds*	8.81	9.52	10.44	12.29	13.80	13.44
Herbicides related compound*	396.49	420.27	435.58	520.01	551.15	539.79
Other hydrocarbons*	325.95	349.47	352.86	430.36	452.31	444.26
Pesticides related compound*	165.95	177.02	181.72	212.87	221.89	214.94
Others*	169.97	179.12	189.61	234.84	249.93	244.28
**Phosphorus***	**750.78**	**796.44**	**820.59**	**973.74**	**1031.33**	**1002.85**
Phytic acid hydrolysis*	93.01	94.59	98.13	118.47	120.81	118.84
Polyphosphate degradation*	489.18	516.27	533.37	635.69	673.60	654.31
Polyphosphate synthesis*	168.59	185.58	189.08	219.57	236.92	229.70
**Secondary metabolism***	**28.00**	**28.82**	**28.56**	**37.96**	**40.94**	**39.14**
Pigments*	**28.00**	**28.82**	**28.56**	**37.96**	**40.94**	**39.14**
**Sulfur***	**1385.80**	**1492.95**	**1536.05**	**1796.76**	**1912.46**	**1866.65**
Adenylylsulfate reductase*	124.83	130.69	138.58	157.37	165.63	160.22

Table S6 (Cont.)

DMSP degradation*	30.05	29.76	31.36		38.70	41.87	40.95
Reduction*	178.23	185.99	194.14		227.08	235.86	231.18
Sulfide oxidation*	91.98	101.84	103.72		119.51	127.48	125.16
Sulfite reduction*	655.05	710.59	730.96		869.99	939.97	915.04
Sulfur oxidation*	195.96	213.11	214.70		246.57	256.96	255.15
Others*	109.68	120.96	122.60		137.53	144.70	138.95
**Virulence***	**303.34**	**316.73**	**327.55**		**376.63**	**392.47**	**389.73**
Antibiotic resistance*	207.81	216.29	224.50		258.20	266.80	265.47
Degradation*	21.17	21.63	20.07		24.43	25.98	25.17
NA*	74.35	78.80	82.98		94.01	99.69	99.09
**Other***	**68.17**	**79.00**	**78.85**		**94.49**	**102.09**	**97.25**
Phylogenetic*	68.17	79.00	78.85		94.49	102.09	97.25

The full name of each sampling site is listed in Figure S2.

Table S7 The relative abundance of functional gene categories as determined by metagenome shotgun sequencing. Numbers in cells are percentages of relative abundance, highest abundances shown in red, middle values in blue and lowest values in white. An asterisk indicates a significant difference between backwater and riverine sites (*t*-test, $P < 0.05$).

	Backwater sites			Riverine sites		
	XXR_E	XXR_M	WJB	XXR_U	BSR_E	SDR_E
Amino acid transport and metabolism	7.54	7.39	8.91	7.32	5.95	7.17
Carbohydrate transport and metabolism	4.73	4.53	4.90	4.76	4.68	4.90
Cell cycle control, cell division, chromosome partitioning	1.07	1.13	1.22	1.09	0.97	1.10
Cell motility	0.21	0.05	0.15	0.45	0.28	0.37
Cell wall/membrane/envelope biogenesis	6.19	4.18	4.59	5.59	5.43	5.66
Chromatin structure and dynamics	0.04	0.15	0.11	0.13	0.16	0.10
Coenzyme transport and metabolism	3.50	3.47	3.74	2.93	2.54	3.12
Cytoskeleton*	0.07	0.92	0.27	0.48	2.05	0.86
Defense mechanisms	1.37	1.22	1.25	1.65	1.76	1.65
Energy production and conversion	6.57	7.90	8.00	5.84	4.95	5.87
Extracellular structures	0.04	0.02	0.02	0.00	0.02	0.03
Function unknown	17.24	12.08	12.03	16.01	15.02	15.67
General function prediction only	13.80	13.34	13.04	14.50	17.58	14.96
Inorganic ion transport and metabolism	4.00	3.53	4.11	4.37	4.11	4.20
Intracellular trafficking, secretion, and vesicular transport	1.65	1.93	1.80	1.94	1.97	2.05
Lipid transport and metabolism	3.28	3.04	3.40	3.30	2.98	3.06
Nuclear structure	0.00	0.00	0.00	0.00	0.01	0.00
Nucleotide transport and metabolism	3.20	3.30	3.43	2.44	2.31	2.60
Posttranslational modification, protein turnover, chaperones	4.04	5.43	4.65	4.75	5.33	4.77
Replication, recombination and repair	7.52	9.40	7.38	7.43	7.74	6.98
RNA processing and modification*	0.06	0.44	0.23	0.22	0.29	0.19
Secondary metabolites biosynthesis, transport and catabolism	1.87	2.00	2.25	1.98	1.72	1.82
Signal transduction mechanisms	1.68	1.76	1.73	2.65	2.52	2.45
Transcription	3.83	3.66	3.95	4.24	3.52	4.00
Translation, ribosomal structure and biogenesis	6.48	9.14	8.83	5.92	6.11	6.43

The full name of each sampling site refers to Figure S2.
